# Asthma and metabolic syndrome: a comprehensive systematic review and meta-analysis of observational studies

**DOI:** 10.34172/jcvtr.2020.20

**Published:** 2020-05-31

**Authors:** Nahid Karamzad, Neda Izadi, Sarvin Sanaie, Elham Ahmadian, Aziz Eftekhari, Mark J.M. Sullman, Saeid Safiri

**Affiliations:** ^1^Student Research Committee, Tabriz University of Medical Sciences, Tabriz, Iran; ^2^Department of Biochemistry and Diet Therapy, Faculty of Nutrition and Food Sciences, Tabriz University of Medical Sciences, Tabriz, Iran; ^3^Nutrition Research Center, Faculty of Nutrition and Food Sciences, Tabriz University of Medical Sciences, Tabriz, Iran; ^4^Student Research Committee, Department of Epidemiology, School of Public Health and Safety, Shahid Beheshti University of Medical Sciences, Tehran, Iran; ^5^Neurosciences Research Center, Aging Research Institute, Tabriz University of Medical Sciences, Tabriz, Iran; ^6^Department of Basic Sciences, Maragheh University of Medical Sciences, Maragheh, Iran; ^7^Department of Social Sciences, University of Nicosia, Nicosia, Cyprus; ^8^Department of Life and Health Sciences, University of Nicosia, Nicosia, Cyprus; ^9^Social Determinants of Health Research Center, Shahid Beheshti University of Medical Sciences, Tehran, Iran; ^10^Tuberculosis and Lung Disease Research Center, Tabriz University of Medical Sciences, Tabriz, Iran; ^11^Rahat Breath and Sleep Research Center, Tabriz University of Medical Sciences, Tabriz, Iran

**Keywords:** Metabolic Syndrome, Prevalence, Asthma, Epidemiology, Meta-analysis

## Abstract

***Introduction:*** This study aimed to perform a meta-analysis on the prevalence of metabolic syndrome (MetS) among patients with asthma and to measure the association asthma has with MetS.

***Methods:*** The Web of Science, Medline, Scopus, Embase and Google Scholar were searched using the "Asthma", "Metabolic Syndrome", "Dysmetabolic Syndrome", "Cardiovascular Syndrome", "Insulin Resistance Syndrome", "Prevalence", "Odds Ratio", "Cross-Sectional Studies", and "Case-Control Studies" keywords. All observational studies reporting the prevalence of MetS among people with and without asthma were included in the study. In the presence of heterogeneity, random-effects models were used to pool the prevalence and odds ratios (OR), as measures of association in cross-sectional and case-control/ cohort studies, respectively.

***Results:*** The prevalence of MetS among patients with asthma (8 studies) and the OR comparing the prevalence of MetS among patients with and without asthma (5 studies) were pooled separately. The pooled prevalence of MetS among patients with asthma was found to be 25% (95% confidence interval (CI): 13%–38%). In contrast, the overall pooled OR for MetS in patients with asthma, compared to healthy controls, was 1.34 (95% CI: 0.91–1.76), which was not statistically significant.

***Conclusion:*** The prevalence of MetS was relatively high in patients with asthma. Furthermore, the odds of MetS was higher in patients with asthma, compared to healthy controls, although this difference was not statistically significant. More original studies among different populations are needed in order to more accurately examine the association between asthma and MetS, as well as the relationship asthma has with the individual components of MetS.

## Introduction

Asthma is one of the most common respiratory ailments and although there are a number of treatments available, these are not always effective for patients with severe clinical symptoms.^1-3^ A recent literature search reported that obesity was an important factor that disrupts the control of asthma symptoms and results in a reduced response to treatment.^4^ Indeed, a robust epidemiological association between asthma and obesity has recently become well established.^5-8^ Asthma has also been associated with other components of metabolic syndrome (MetS), such as hypertension and insulin resistance, irrespective of elevated body mass.^8-11^ According to reports from the World Health Organization (WHO), obesity has shown a drastic increase in recent decades. For instance, more than one-third of the American population were estimated to be obese from 2009 to 2010.^12^ Obesity is a major risk factor for several other diseases, such as: diabetes, arthritis, cardiovascular problems, obstructive sleep apnea and cancer.^13^ Also, as shown in several cross-sectional studies, obesity has a strong relationship with asthma,^14^ as a predisposing factor.^15^ Moreover, obesity is an obstacle in asthma control and treatment, and weight loss has been associated with improved clinical symptoms of asthma and lung function.^7,16,17^ The cellular mechanism by which obesity affects asthma is still under debate.^18^ Although inflammatory cell infiltration occurs in obesity, it has not been reliably observed in those suffering from asthma and so other mechanisms, such as hyperinsulinemia, dyslipidemia and hyperglycemia should be taken into account, rather than solely focusing on the inflammatory condition.^19^

MetS is characterized as a syndrome in which three of the following symptoms exist: disturbed fasting glucose metabolism, dyslipidemia, obesity and hypertension.^20-22^ Also, increased risk of coronary heart disease, atherosclerosis and diabetes have a direct link with MetS.^21-23^ Hepatic failure, sleep apnea and chronic inflammatory states, including asthma, are also common clinical signs of MetS.^21-23^ Although the association between asthma and MetS has been examined in a number of studies, to the best of our knowledge no meta-analysis has investigated the association between asthma and MetS. Thus, we aimed to conduct a meta-analysis to examine the association between asthma and MetS using published observational studies.

## Materials and Methods

### Search strategy and study selection

This meta-analysis was conducted using the “Preferred Reporting Items for Systematic Reviews and Meta-Analysis” (PRISMA) guidelines.^24^ All cross-sectional, case-control and cohort studies reporting the prevalence of MetS among people with and without asthma were included in the study.

The following keywords were identified using the medical subject headings (MESH) in Medline: “Asthma”, “Metabolic Syndrome”, “Dysmetabolic Syndrome”, “Cardiovascular Syndrome”, “Insulin Resistance Syndrome”, “Prevalence”, “Odds Ratio”, “Cross-Sectional Studies”, and “Case-Control Studies”. These keywords were used to search the following databases: Web of Science, Medline, Scopus, Embase and Google Scholar.

### Inclusion and exclusion criteria

All English language articles that were published from inception to June 2018 were imported into Endnote X6. Firstly, the title and abstracts of all identified papers were examined and then the full-texts of all relevant articles were examined. The full-text articles were then meticulously examined by two researchers trained (NI and SS) using the specified criteria and any disagreements were resolved by consulting a third researcher (NK).

The location of the study, study design, time, sampling procedures and statistical analyses were also examined in the selection process. Non-original articles, such as reviews, were excluded but research letters published in highly prestigious journal were included. Moreover, all studies on people suffering from other diseases were excluded. Finally, an e-mail was sent to the corresponding author of any articles which could not be included in the meta-analysis, due to missing details.

### Data extraction and quality assessment

The data extraction was conducted by the two aforementioned researchers and familiar with data extraction based on formulated a research question i.e. using the PICO model (NI and SS) and any disagreements were resolved by consulting the previously mentioned third researcher (NK). The following variables were extracted for the analyses: study specifications (first author’s name, study design, location and date of publication), participant details (age, sample size, and each study groups’ health status, in relation to asthma and MetS). The quality of reporting in each study was examined using the STROBE checklist (22 items) and only those which reached the minimum acceptable level of quality (>15 items) were included in the analysis stage ^25^. The Newcastle-Ottawa tool^26^ was also used to examine the risk of bias in the included studies, where the minimum and maximum scores possible were 0 and 9 for case-control and cohort studies and 0 and 10 for cross-sectional studies. Studies with scores ≥6, 3<score<6, and score <3 were considered to be *low risk* , *moderate risk* and *high risk,* respectively. This tool examined the risk of bias in three aspects (*selection* , *comparability* and *outcome/exposure*) for the cross-sectional, case-control and cohort studies included in the current meta-analysis.

All researchers involved in searching different databases, data extraction and quality assessment had previous experience in conducting or collaboration in a meta-analysis study.

### Statistical analysis

A random-effects model was used to pool the OR of MetS among patients with and without asthma, to identify heterogeneity among the studies. Heterogeneity between the studies was determined using the I^2^ index. Meta-regression for different variables including age, study design, MetS diagnostic criteria and publication date were used to determine the source of heterogeneity, when the I^2^ index was found to be higher than 0.6. In addition, sensitivity analysis was perform to explore the impact of excluding or including studies in analysis based on sample size, MetS diagnostic criteria and study design. Finally, publication bias was examined using a Funnel plot and Egger’s test.^27^ All statistical analyses were performed using Stata software version 13 (Stata Corp, College Station, TX, USA).

## Results

**Figure 1:**
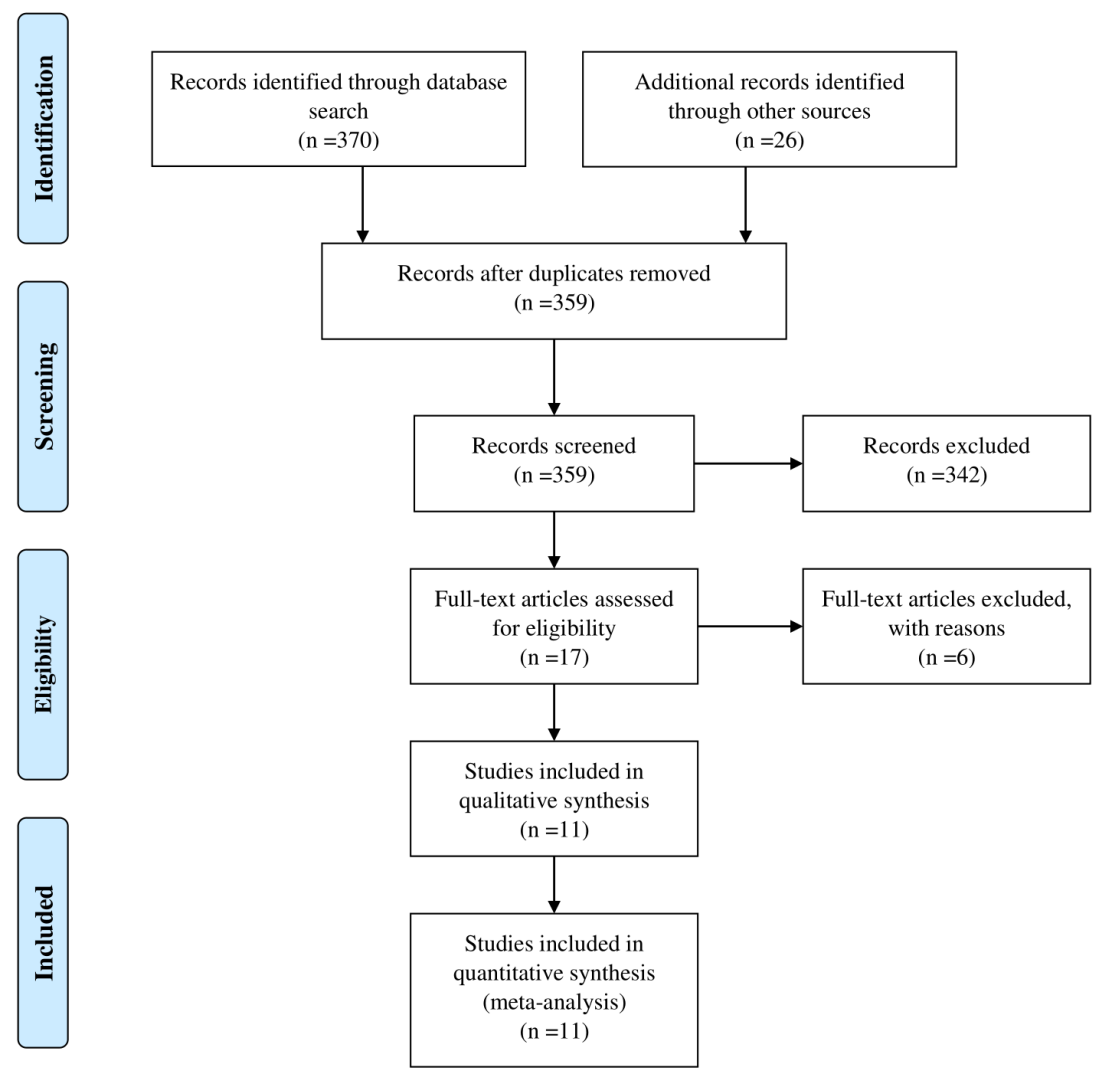
Follow diagram of the systematic review & meta-analysis of asthma and MetS.

The process of article selection is illustrated in Figure 1. This shows that 370 studies were identified at the first stage of the search process and an additional 26 articles were found through searching the reference lists of these articles. Thirty-seven articles were excluded due to duplication of the data. Following a review of all remaining abstracts, 342 papers were excluded due to duplication (n=4) and irrelevant data (n=338) and the full text versions of 17 articles were downloaded for detailed analysis. Following the assessment of full-texts, six papers were excluded due to inappropriate study design (n=2), duplication (n=1) and irrelevant data (n=3). Finally, seven cross-sectional studies, one case-control study and three cohort studies were considered for the final stage of the study. However, only five studies had information regarding the association between MetS and asthma using an OR. All studies included in this systematic review and meta-analysis were published in English during the period January 2010 to June 2018, and the ages of the participants ranged from 12-78 years old. More detailed information regarding the studies included in this systematic review and meta-analysis are provided in Table 1. The results of the risk of bias assessment for the studies included in the meta-analysis are presented in Appendix Table 1.

**Table 1:** Eligible studies reporting the prevalence of metabolic syndrome in patients with asthma

**First Author**	**Country**	**Study Design**	**MetS Criteria**	**DOP**	**Age Range**	**Mean Age**	**Group**	**Gender**	**Sample Size**	**N.**	**MetS Prev. (%)**
Forno^29^	U.S.	Cross-Sectional	Three of the five criteria: fasting glucose ≥ 110 mg/dL; WC ≥ 75th percentile; fasting TG ≥ 100 mg/dl; HDL ≤ 50 mg/dL; and SBP ≥ 90th percentile	2015	12-17	-	-	Both	1429	224	15.7
								Male	745		
								Female	684		
							No asthma	Both	1334	58	9.02
							Asthma	Both	95	7	15.77
Aydin^30^	Turkey	Case-Control	Modified WHO diagnostic criteria	2013	Postmenopausal	Asthma=57.5±13.9/Control=59.6±12.8	-	Female	75	12	26.7
Assad^28^	U.S.	Longitudinal Analysis	ATP-III criteria	2013	-	24.9±3.6 (Male=24.6±3.6/ Female= 25.1±3.7)	No asthma	Both	4017	88	2.2
								Male	1900	53	2.8
								Female	2117	36	1.7
							Asthma	Both	602	19	3.2
								Male	185	3	1.6
								Female	417	16	3.8
Ahmed^31^	Pakistan	Cross-Sectional	-	2016	-	-	-	Both	154	46	29.87
								Male	80		
								Female	74		
Adeyeye^9^	Nigeria	Cross-Sectional	The international diabetes Federation (IDF) criteria	2012	14-78	46.5±17.2 (Male= 44.9±16.2/ Female=47.6±17.7)	-	Both	158	28	17.7
								Male	63	13	20.6
								Female	95	15	15.8
Uzunlulu^32^	Turkey	Case-Control	The international diabetes Federation (IDF) criteria	2011	-	Asthma=43.83±10.98 Control= 42.01±9.21	No asthma	Both	98	33	33.7
								Male	17	6	35.3
								Female	81	27	33.3
							Asthma	Both	90	33	36.7
								Male	20	6	30
								Female	70	27	38.6
Pantoja-Alcantar^33^	Mexico	Cross-Sectional	ATP III criteria	2012	-	-	-	Both	39	11	28.2
Singh^34^	India	Cross-Sectional	The NCEP ATP III definition	2016	17-59	-	-	Both	60	20	33.3
								Male	28		
								Female	32		
Del-Rio-Navarro^35^	Mexico	Cross-Sectional	de Ferranti criteria	2010	Adolescent	-	-	Both	174	72	41.34

### The association between MetS and asthma

**Figure 2:**
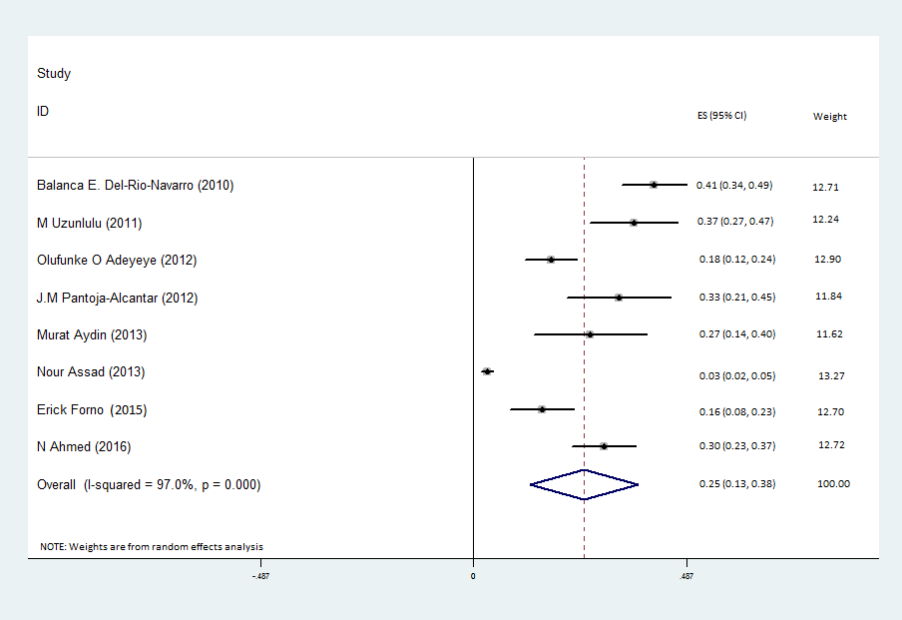
Forest plot of metabolic syndrome prevalence among patients with asthma.

**Figure 3:**
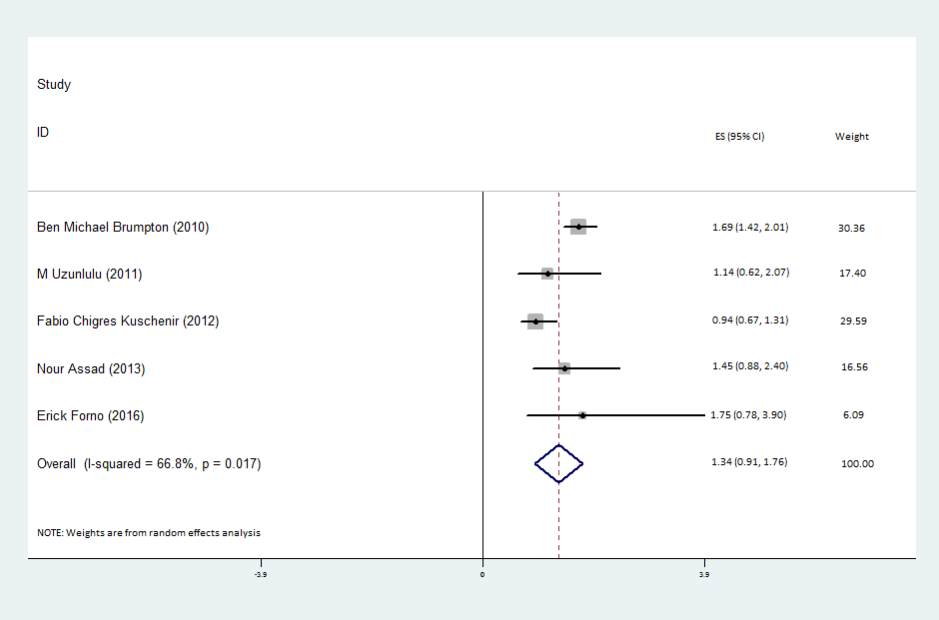
Forest plot of metabolic syndrome odds among patients with asthma, compared with healthy controls.

The prevalence of MetS was pooled across the 8 studies and was estimated to be 25% (95% confidence interval (CI): 13–38%) among patients with asthma (Figure 2).Furthermore, the association between MetS and asthma was examined across the 5 studies with ORs, which found that the odds of MetS was 34% higher in patients with asthma, compared to a healthy control group, but this was not statistically significant [odds ratio (OR): 1.34; 95% confidence interval (CI): 0.91-1.76] (Figure 3). More detailed information about the studies comparing the prevalence of MetS between patients with and without asthma are presented in Table 2.

**Table 2:** Prevalence of metabolic syndrome in patients with asthma compared to healthy controls

**First Author**	**DOP**	**Study Design**	**Assessment of Asthma**	**Country**	**Age Range**	**Mean Age**	**Type of Effect**	**Gender**	**Group**	**Effect** **(95% CI)**
Kuschnir^36^	2018	Cross-sectional	Self-administered questionnaire	Brazil	12-17	14.6±1.6	Prevalence Ratio	Both	Current Asthma	0.94 (0.67-1.31)
									Severe Asthma	2.43 (1.39-2.27)
Forno^29^	2015	Cross-sectional	Diagnosed by a doctor or other health care professional/Spirometry	U.S.	12-17	-	Crude OR	Both	Current Asthma	1.75 (0.78-3.90)
Brumpton^37^	2013	Prospective cohort	Self-administered questionnaire	Norway	19-55	-	Adjusted OR	Both	Current Asthma	1.57 (1.31-1.87)
Assad^28^	2013	Longitudinal analysis	Self-reported presence of asthma symptoms	U.S.	-	24.9±3.6 (Male=24.6± 3.6/ Female= 25.1±3.7)	Crude OR	Both	Current Asthma	1.45 (0.88-2.40)
								Male		0.57 (0.18-1.83)
								Female		2.25 (1.25-4.03)
Uzunlulu^32^	2011	Case-control	Diagnosed by a pulmonologist	Turkey	-	Asthma= 43.83±10.98 Control= 42.01±9.21	Crude OR	Both	Current Asthma	1.14 (0.62-2.07)
								Male		0.78 (0.19-3.17)
								Female		1.30 (0.66-2.53)

### Heterogeneity and meta-regression

The I^2^ statistics was used to assess heterogeneity across the studies. A statistically significant level of heterogeneity was found across the studies estimating the prevalence of MetS among patients with asthma ((I^2^=97.0%, *P*  = 0.001) (Figure 2). Meta-regression was then used to find the source of the heterogeneity among the studies, which found that age (*P*  = 0.61), study design (*P*  = 0.19), MetS diagnostic criteria (*P*  = 0.12) and publication date (*P*  = 0.29) were not responsible for the reported heterogeneity. Moreover, statistically significant heterogeneity was found for the estimated association between MetS and asthma status (I^2^=66.8%, *P*  = 0.017) (Figure 2). Meta-regression of this data showed that age (*P*  = 0.43), study design (*P*  = 0.98), and publication date (*P*  = 0.99) were not responsible for the observed heterogeneity. As the aforementioned variables were not significant, no subgroup-analysis was conducted. Sensitivity analysis showed that there was no influential study across the studies estimating the associations between MetS and asthma status.

### Publication bias

**Figure 4:**
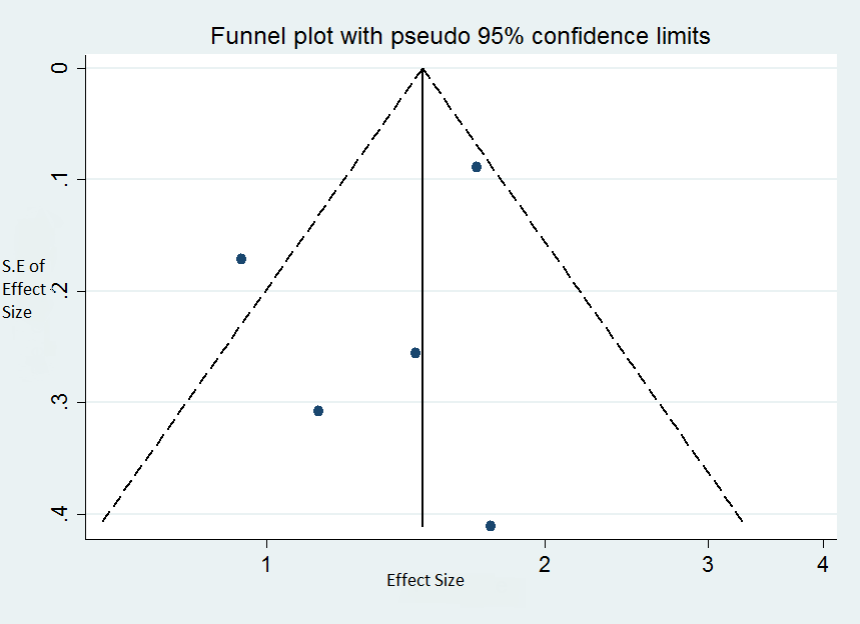
Funnel plot assessing publication bias on the association between metabolic syndrome and asthma status.

No publication bias was found for the association between MetS and asthma status (Egger’s test: *P*  = 0.46). The corresponding Funnel plot is shown in Figure 4.

## Discussion

The present study revealed that the prevalence of MetS was a lot higher (25%) in patients with asthma, meaning that this syndrome needs to be addressed and controlled in patients with asthma. On the other hand, although the present study found that the odds of being diagnosed with MetS were higher in patients with asthma, compared to healthy controls, this association was not statistically significant. However, these results should be interpreted with caution as the number of published studies included in the meta-analysis was not high and there may not have been sufficient statistical power to adequately examine the association between MetS and asthma. Furthermore, the associations asthma had with the components of MetS were not reported in these studies. This is problematic, as asthma may have completely different relationships with the different components of MetS. In addition, according to the research evidence, obesity is just one of the building blocks of MetS which increases the risk of asthma.^38^

Several mechanisms may be responsible for the relationship between asthma and MetS, such as localised and systemic inflammation, oxidative hazard and insulin resistance. System and airway inflammation have previously been identified as having a key role in the relationship between obesity and asthma. Enhanced pro-inflammatory cytokines, such as leptin, tumor necrosis factor α and interleukins are secreted from the adipose tissues of obese patients with asthma, which triggers further systemic inflammatory response.^39,40^ Macrophage proliferation and the differentiation of lung tissue are also plausible consequences of airway inflammation.^39^ However, clinical investigations have not supported these hypotheses.^41^ Another mechanism which has previously been mentioned is that insulin resistance may lead to airway dysfunction, since the association between asthma and insulin resistance has also been reported in a number of different studies.^8,42^ The reduction of glucose consumption and the induction of abnormal fat metabolism in muscles, which is concomitant with impaired mitochondrial energy production, may also contribute to skeletal muscle respiratory resistance. Therefore, respiratory muscle malfunction may lead to the airflow impediment observed in asthma.^43^ Regarding oxidative stress, the enhanced production of systemic reactive oxygen species (ROS) has been observed in obese patients, while asthmatics show low levels of endogenous antioxidants.^44,45^ However, it is not yet clear whether airway oxidative stress occurs as a consequence of systemic ROS formation, due to obesity.^39^

A number of regional and age-group studies have found asthma to have a relationship with MetS and its clinical manifestations, such as hypertension and hypertriglyceridemia.^47-49^ However, in a study conducted by Assad et al no significant link between asthma and MetS was found, after adjustments for BMI were made.^28^ Thus, it remains unclear whether those with MetS who have a normal weight also have an elevated risk of asthma. Even in vivo studies using animals have not produced conclusive results.

Lipoprotein dysfunction reported in asthma pathogenesis, suggests the use of statins could be beneficial in these patients, although there is still some debate on this issue.^49-51^ In addition to cholesterol metabolism regulating the effects of statins in asthmatics, the anti-inflammatory properties of these drugs have been thought to have a role in asthma control.^44,52-54^ In particular, Lovastatin has been shown to diminish the differentiation and proliferation of asthmatic bronchial fibroblasts in vitro.^55^ Furthermore, Atorvastatin and Simvastatin attenuate the number of inflammatory cells found in saliva specimens.^28^ In addition, bisphosphonate, such as alendronate, have been shown to provide protection against asthma via reducing eosinophilic airway inflammation by reducing the regulation of several cytokines.^56^ Indeed, the role of diet and exercise should not be overlooked in the treatment of asthmatic patients who are overweight or obese.^21,57^ Supplements such as retinoids, retinoic acid and fenretinide exert their beneficial effect by reducing the inflammatory response.^58,59^ While it is clear that these agents can reduce bronchial obstruction, more research is needed to ascertain the potential protective impacts of novel therapies to control MetS in asthmatic patients, since these may be useful in treating non-responding or uncontrolled asthma.^60,61^ Chemokines and their receptors have been shown to be involved in adipose tissue enlargement and the initiation of insulin resistance.^62,63^ Thus, the inhibition of chemokine receptors could potentially be helpful in asthmatic patients with MetS.^64^

### Limitations of the study

Although the present study is the first meta-analysis to investigate the association between MetS and asthma, there are some limitations that should be considered when interpreting the results. Firstly, the number of studies included was less than 10 and the power was insufficient to examine publication bias. In addition, the source of heterogeneity could not be identified in the meta-regression due to a lack of statistical power and neither could subgroup-analysis be undertaken. Finally, more original research is need in different populations and among different age and sex groups, in order to increase the generalizability of any future meta-analyses.

## Conclusion

The current meta-analysis found the prevalence of MetS was relatively high in patients with asthma. Furthermore, the odds of MetS was higher in patients with asthma than that found among healthy controls, although this difference was not statistically significant. More original studies in different populations are needed to examine the association asthma has with MetS, as well as the relationship that asthma has with the individual components of MetS.

## Acknowledgments

We would like to thank Social Determinants of Health Research Center of Shahid Beheshti University of Medical Sciences for financial support.

## Competing interests

None to declared.

## Ethical approval

Not applicable.

## Funding

The present study was supported by Social Determinants of Health Research Center, Shahid Beheshti University of Medical Sciences, Tehran, Iran (grant No. 20974).
